# Developing the better and effective nursing education for improving transcultural nursing skills cultural competence and cultural sensitivity assessment tool (BENEFITS-CCCSAT)

**DOI:** 10.1186/s12912-023-01476-6

**Published:** 2023-09-27

**Authors:** Ayla Yava, Betül Tosun, Katalin Papp, Valérie Tóthová, Eda Şahin, Emel Bahadir Yılmaz, Ezgi Dirgar, Věra Hellerová, Sandra Tricas-Sauras, Mirko Prosen, Sabina Ličen, Igor Karnjus, M. Dolors Bernabeu Tamayo, Juan M. Leyva-Moral

**Affiliations:** 1 Faculty of Health Sciences, Nursing Department, Hasan Kalyoncu University, Gaziantep, Turkey; 2 Nursing Department, Faculty of Health Sciences, University of Debrecen, Nyíregyháza, Hungary; 3 Faculty of Health and Social Sciences, Institute of Nursing, Midwifery and Emergency Care, University of South Bohemia, České Budějovice, Czech Republic; 4 Faculty of Health Sciences, Nursing Department, Giresun University, Giresun, Turkey; 5 Gaziantep University, Faculty of Health Sciences, Department of Midwifery, Gaziantep, Turkey; 6 Department of Healthcare, Design and Technology, Erasmus Hogeschool Brussel, Brussels, Belgium; 7 Social Approaches to Health Research Centre, School of Public Health, Université Libre de Bruxelles (ULB), Brussels, Belgium; 8 Faculty of Health Sciences, Department of Nursing, University of Primorska, Polje 42, Izola, 6310 Slovenia; 9 Faculty of Medicine, Nursing Department, Universitat Autònoma de Barcelona, Barcelona, Spain

**Keywords:** Transcultural nursing, Cultural competency, Cultural sensitivity, Students, Nursing

## Abstract

**Background:**

A clear need for the development of new comprehensive, reliable, sensitive and valid measurement tools to adequately asses the cultural competence and cultural sensitivity of nursing students exists. This study aimed to develop a new measurement tool to assess the nursing students’ cultural competence and sensitivity.

**Methods:**

This cross-sectional, instrument development study’s first phase included postgraduate nursing students (n = 60) for the piloting study, and the second one included undergraduate nursing students (n = 459) for the main survey. This study used two data collection forms: The Student Descriptive Information Form and the Better and Effective Nursing Education for Improving Transcultural Nursing Skills Cultural Competence and Cultural Sensitivity Assessment Tool (BENEFITS-CCCSAT) draft. The content validity index was calculated using the Davis method. Cronbach’s α coefficient and the item total correlation were calculated during the reliability analysis. The Kaiser-Meyer-Olkin (KMO) coefficient test, Bartlett significance test, and explanatory factor analysis (EFA) were used to evaluate the validity of the assessment tool.

**Results:**

Scale validity and reliability analyses showed that the BENEFITS-CCCSAT included 26 items and five sub-dimensions: respect for cultural diversity; culturally sensitive communication; achieving cultural competence; challenges and barriers in providing culturally competent care; and perceived meaning of cultural care.

**Conclusion:**

The BENEFITS-CCCSAT appears to be a valid and reliable instrument for measuring the cultural sensitivity and cultural competence of nursing students. This can be of great use, especially before attending clinical areas, and can offer both students and faculty reliable information to promote reflective and critical thinking, especially in areas where improvement is needed.

## Introduction

According to the 2020 United Nations World Immigration Report, there were 272 million immigrants worldwide in 2019, comprising 3.5% of the global population [1]. Immigrating individuals and families may experience medical problems that negatively affect their physical, emotional, and social wellbeing [2]. Moreover, globalization facilitates many changes in medical care by integrating economic, social, political, and cultural links among different regions of the world. To promote the cohabitation of different cultures, it is important that medical professionals are equipped with the cultural knowledge and skills required to meet the needs of these communities [3].

Additionally, it must be considered that people who migrate often may enter new territory and face new, unfamiliar social relationships. Cultural differences and prejudices can be a source of conflict for both the patient and their nurse. In the healthcare context, this could be managed by identifying the appropriate level of nursing cultural competencies and developing tailored and adequate educational programs that may ameliorate those competencies [4]. Nurses must provide culturally competent care; therefore, it is crucial to equip nursing students with the necessary knowledge and skills in transcultural nursing care [5, 6]. For this reason, international nursing scholars have underlined the importance and challenges regarding educational interventions that contribute to increasing cultural competence. Also, the need to develop specific curricula and training programs that promote cultural knowledge, awareness, and culturally sensitive nursing skills among students is underlined. These strategies should be integrated in formal education programs with differences in identified targets, curriculums, educational interventions, and assessment methods [7–9]. In one systematic review that analyzed the relationship between treated patient outcomes and educational strategies for promoting medical professionals’ cultural competence, it was reported that the assessment methods and results of the education programs mentioned were not sufficient. Moreover, due to the various educational strategies and assessment methods used in these studies, it was difficult to identify the effects of the strategies and no clear results were found [3]. Tosun et al., (2021) indicated that a limited number of studies have generally proven the effectiveness of cultural nursing education. The study noted that teaching methods and program duration differ widely despite the educational content. Researchers generally evaluate the educational programs using similar or the same measurement tools. More sensitive, comprehensive, and alternative measurement tools should be developed to evaluate transcultural nursing education programs [10].

### Background

The topics of transculturalism and multiculturalism have been introduced into the current nursing education curriculum in various countries. This process also demands the use of new educational strategies, forms, and methods focused on the acquisition, development, and strengthening of students’ cultural competences [11].

Transcultural nursing education is essential, as well as how and by which methods this education will be evaluated [7, 12]. Various tools to evaluate the effectiveness of the training have been reported [7–9]. In Majda et al.’s (2021) assessment of the cultural competence and cultural intelligence of master’s degree nursing students, the “Cross-Cultural Competence Inventory and Cultural Intelligence Scale” were used [11]. In the study by Choi and Kim (2018), the “Cultural Competence Scale for Nursing Students” was used [5]. Some of these tools were developed by nursing theorists who used their own cultural competence models. For instance, Campinha-Bacote developed two assessment instruments, the “Inventory for assessing the process of cultural competence among healthcare professionals” and its student version [13, 14], while Papadopoulos et al. (2008) developed the “Cultural Competence Assessment Tool” [15]. Some assessment instruments, such as the “Transcultural Self-Efficacy Tool” and its adapted evaluation tool by Jefferys (2010) [16], and the “Eldercare Cultural Self-Efficacy Scale” [17] are grounded in self-efficacy psychology theory. Many researchers use their own questionnaires combined with or without these structured scales [5, 9, 18]. For this reason, it has been suggested that there is a need for a new assessment tool based on several nursing models for evaluating nursing students’ health promotion and the effectiveness of cultural competence and cultural sensitivity training programs. Therefore, this study focused on the development of a new comprehensive tool to evaluate nursing students’ cultural competence and cultural sensitivity.

## Method

### Design

This study developed an instrument on cultural competence and its psychometric analysis.

### Setting

Research was conducted between January-April 2021, and it included a sample of undergraduate third-year and fourth-year nursing students at one state university and one foundation university located in north of Turkey and south of Turkey, respectively. At these universities, undergraduate nursing education comprises eight semesters after the completion of high school.

### Study sample

This study was performed in two stages: a pilot study and a main survey. During the pilot phase, when the item pool was created, the study comprised graduate nursing students from the aforementioned universities (n = 98). The data collection form and draft tool were delivered online to 70 postgraduate nursing students who volunteered to participate in the test and retest analysis fifteen days (2 weeks) after they were created. The analysis was completed with a sample group of 60 postgraduate students who fully completed the retest scale.

In accordance with the principle that scale development studies should include a sample size that is at least five to ten times larger than the number of items on the measurement tool [19–21], the sample size was estimated at 480 nursing students (48 items of the draft tool; 10 × 48 = 480 students). The inclusion criteria for nursing students were knowledge of fundamental nursing skills and interventions, completion of at least two semesters of clinical practice, third or fourth-year nursing student status, and voluntary participation in the study. During this phase of the study, the data collection tool was delivered online to all undergraduate nursing students (N = 481). As a result, 462 responses were received from these nursing students. As three participants were excluded from the study because of incomplete data collection forms, the study ultimately included 459 nursing students. The response rate during this study was 95% (the sample size was 9.5-times the number of items on the scale).

### Data collection

The online data collection forms were created using Google Forms and delivered to the social media accounts and e-mail addresses of the nursing students. An informed consent form was placed at the top of the data collection forms, and participants were asked to place a checkmark in a box if they agreed to voluntarily participate in the study. Completion of the data collection forms required nearly 20 min for each participant. The assessment tool’s initial version was revised after the pilot study involving graduate nursing students. The validity and reliability tests were executed among third- and fourth-year students working towards their bachelor’s degree in nursing. The final version of the tool of the data collection and informed consent forms were also delivered online to nursing students working towards their bachelor’s degree.

### Data collection forms

This study used two data collection forms: The Student Descriptive Information Form and the Better and Effective Nursing Education for Improving Transcultural Nursing Skills (BENEFITS) Cultural Competence and Cultural Sensitivity Assessment Tool (BENEFITS-CCCSAT) draft. The Student Descriptive Information Form included 10 questions that collected the sociodemographic characteristics of the nursing students, their native language, other languages spoken, and whether they previously cared for a patient from a different culture. To develop the BENEFITS-CCCSAT, 14 nursing scholars from Turkey, Spain, the Czech Republic, Slovenia, Hungary, and Belgium who have experienced and studied transcultural nursing education published a systematic review study10 on the effectiveness of transcultural nursing education. Based on this article the item pool was established for the assessment tool. The item pool was developed in English and included 53 items; language validity and content validity analyses were performed with the opinions of seven experts. After the expert opinions were considered, the draft scale was revised; it now included 47 items. Pretesting was performed among 60 nursing students with graduate degrees. The final draft of the scale for the main survey included 7 Likert-type, 35 positive, and 8 negative (total of 43) scoring items. Negative items were reverse-coded during the analysis phase. High scores obtained from the scale indicated that nursing students had high cultural competence, cultural sensitivity, and transcultural nursing skills.

### Statistical analyses

Study data were analyzed using the “IBM Statistical Package for Social Sciences (SPSS©) for Windows version 26.0”. The numbers, percentages, means, and standard deviations were calculated during the descriptive analysis. The Shapiro–Wilk test and Skewness and Kurtosis test were used to analyze normality. The content validity index was calculated using the Davis method. The Wilcoxon test and interclass correlation calculations were used for the test-retest comparison during the pilot study. Cronbach’s α coefficient and the item total correlation were calculated during the reliability analysis. The reliability of the measurement model was also tested using the average explained variance (AVE) and composite reliability (CR) values for each factor separately. The Kaiser-Meyer-Olkin (KMO) coefficient test, Bartlett significance test, and explanatory factor analysis (EFA) were used to evaluate the validity of the assessment tool. Statistical significance was considered when *p* < 0.05.

### Language adaptation

As the researchers were members of a multinational project team and had different native languages, a common language (English) was used to develop the item pool (53 items) for the draft tool. Group translation and retranslation methods were used for language adaptation [22, 23]. Using the group translation method, two native Turkish-speaking translators with excellent command over the English language and a native English-speaking translator with excellent command over the Turkish language translated the item pool from English to Turkish. Then, the researchers, a linguistic expert, and translators assessed and finalized the tool items together. Retranslation of the tool was performed by two native Turkish-speaking translators with excellent command over the English language and native English-speaking translators with excellent command over the Turkish language. After retranslation, the tool item pool was compared with the original item pool. The Turkish version of the item pool (48 items) was prepared by five Turkish researchers from the project team and delivered to seven experts for their opinion. After receiving the experts’ opinions (47 items) and completing the pilot study (43 items), the final draft version of the tool was created, and language adaptation was completed.

### Content validity

To determine the study’s content validity, the draft tool (48 items) was delivered to seven experts who evaluated the clarity of the items and their relevance to the subject. The experts used the Davis method to form their opinions. Experts were asked to assess the items as “appropriate,” “appropriate but needs minor revision,” “needs major revision,” or “inappropriate.” According to the Davis method, items with a content validity index less than 0.80 should be excluded [24]. Therefore, one item, “I think that attitudes of majority groups are one of the important factors that affect the behaviors of minority groups,” with a content validity index of 0.54 was excluded from the draft tool and five items were revised. The final draft of the tool had 47 items and was used for the pilot study.

### Ethical considerations

Before starting the study, ethical approval was obtained from the XXX Faculty of Health Sciences Non-Invasive Research Ethical Board, and all permissions were received from the universities where the study was conducted (Date: 19 January 2021, Decision No: 001). Data collection was performed after ethical approval and legal permission was received after the participants provided informed consent.

## Results

### The pilot study

During the pilot study, total of 60 nursing students across two universities who were enrolled in graduate degree education programs and had not attended any classes regarding transcultural nursing care were tested and retested to determine the reliability of the tool and the clarity of the included items 2 weeks after the draft tool was created. Participants were asked to complete the forms and to note whether they clearly understood each item of the draft tool for the pilot study. The Wilcoxon test did not reveal any significant differences between the test and retest mean scale scores (z = − 1.750; *p* = 0.080). Upon assessment of the test and retest responses to each item, the difference between the test and retest mean item scores was statistically significant for some items (*p* < 0.05). Additionally, it was found that some items were not clearly understood. Therefore, four items were excluded from the tool and six items were revised by the researchers to make them clearer before transitioning to the tool validity and reliability assessment phase. The intraclass coefficient for the average measures was 0.951 (95% confidence interval, 0.918–0.971; *p* < 0.001), and the reliability coefficient for the overall scale was α = 0.953. Finally, a draft tool including a total of 43 items was prepared for the main survey validity and reliability analyses.

### Main survey of the study

The mean age of the students was 22.14 ± 1.53 years (minimum–maximum, 19–32 years), 78.6% were female, and almost all (97.2%) were born in Turkey. Most of the students (83.7%) spoke Turkish as their native language, 13.7% spoke Kurdish, and 2.6% spoke Arabic. Almost all the students (99.1%) confirmed having religious beliefs, 22.7% of them had work experience, and almost half (44.2%) provided care in clinical practice to patients from a different culture.

The KMO test was performed to assess the suitability of the sample size for the factor analysis before the EFA was performed. The KMO value was calculated as 0.883. According to the Bartlett sphericity test results, χ2 was 9683.680 (df = 325; *p* < 0.01).

EFA was performed to reveal the factor pattern of the multidimensional BENEFITS-CCCSAT tool comprising 43 items. Items 6, 15, 16, 17, 18, 19, 20, 21, 22, 23, 24, 25, 26, 27, 28, 29, and 39 were excluded from the tool. As a result of the EFA, the BENEFITS-CCCSAT was revised to have 26 items grouped into five dimensions: “respect for cultural diversity,” “culturally sensitive communication,” “achieving cultural competence,” “challenges and barriers in providing culturally competent care,” and “perceived meaning of cultural care.”

The essential components analysis as the factoring method and Varimax, a vertical rotation method, were used to inspect the factor pattern of the BENEFITS-CCCSAT. An acceptance level of 0.40 was identified for each factor load value in the analysis to reveal the factor pattern of the tool. The results of the Varimax rotation showed that the five-factor structure was suitable for the items, and the items were organized into the five-factor structure during the analysis. These factors explained 69.826% of the variance (Table 1).

**Table 1:** Results of the explanatory factor analysis of BENEFITS-CCCSAT

Dimensions		Rotated Factor Loads*	Explained Variance	Eigen Value
**Perceived meaning of cultural care**			18.664	8.471
Item42	I consider the involvement of individual members of a multidisciplinary team in the care of a patient with a different religion important.	0.820		
Item43	I consider the involvement of individual members of a multidisciplinary team in the care of a patient from a different ethnic group important.	0.808		
Item41	I consider the involvement of individual members of a multidisciplinary team in the care of a patient from a different culture important.	0.805		
Item40	I consider meeting people of different religions important for gaining cultural competence.	0.757		
Item38	I perceive meeting a person from a different culture (even during care provision) as an opportunity to develop my cultural competence.	0.704		
Item37	I perceive meeting a person of a different religion (even during care provision) as an opportunity to develop my cultural competence.	0.677		
Item30	I consider it important to try to communicate with patients in their native language.	0.590		
Item31	I try to perceive the requirements and particularities related to the protection of privacy during the provision of care to persons of a different ethnic group.	0.505		
**Culturally sensitive communication**			16.482	5.049
Item34	I think it is important to find an interpreter for a patient who is unable to speak my language.	0.900		
Item33*	I have concerns about communicating with the family of a patient with a different religion.	0.876		
Item35*	I have concerns about integrating the family of a patient of a different culture into the overall process of care provision.	0.865		
Item32*	I have concerns about communicating with the family of a patient from a different cultural background.	0.847		
Item36*	I have concerns about integrating the family of a patient with a different religion into the overall process of care provision.	0.839		
**Respect for cultural diversity**			15.833	1.771
Item3	I respect the needs of patients from different cultural backgrounds.	0.867		
Item2	I respect the needs of patients with different religious beliefs.	0.853		
Item1	I respect the needs of patients from different ethnic groups.	0.841		
Item7	I consider lifelong education in transcultural nursing to be important for nurses.	0.682		
Item4	I think that the provision of culturally adequate care is indispensable for healthcare systems in the current world.	0.616		
Item5	I think that transcultural nursing education extends/increases competency for providing culturally sensitive care.	0.585		
**Challenges and barriers in providing culturally competent care**			10.368	1.552
Item10*	It is challenging for me to provide care to a patient from a different ethnic group.	0.836		
Item9*	It is challenging for me to provide care to a patient with a different religion.	0.788		
Item8*	It is challenging for me to provide care to a patient from a different cultural background.	0.767		
Item11*	I have concerns about culturally competent care.	0.611		
**Achieving cultural competence**			8.514	1.328
Item13	I understand the concept of “cultural competence.”	0.806		
Item12	I understand the concept of “transcultural nursing.”	0.789		
Item14	I am capable of using cultural competence during the nursing process.	0.746		
**Total Explained Variance = 69.862**				

*Note.* *Negative items were reverse-coded

The reliability coefficient (Cronbach’s α) of the whole BENEFITS-CCCSAT tool was 0.828, and it varied from 0.789 to 0.942 for the dimensions. Additionally, the corrected item total correlation values for the reliability of the scale were between 0.482 and 0.892 (Table 2).

**Table 2:** Distribution of scores and internal consistency of the BENEFITS-CCCSAT and its dimensions

	Items	Theoretical Min-Max	Mean±SD	Cronbach’s α
**BENEFITS-CCCSAT**	26	26–182	132.86 ± 14.56	0.828
Perceived meaning of cultural care	8	8–56	46.16 ± 6.26	0.891
Culturally sensitive communication	4	4–28	11,99 ± 5.49	0.862
Respect for cultural diversity	6	6–42	37.91 ± 4.14	0.897
Challenges and barriers in providing culturally competent care	5	5–35	19.79 ± 8.06	0.942
Achieving cultural competence	3	3–21	17.00 ± 2.77	0.789

*Note.* Max, maximum; Min, minimum; SD, standard deviation

To determine the distinctiveness of the tool items, the raw scores were ranked from largest to smallest, and the mean scores of the lower group (lower 27%) and upper group (upper 27%) were compared using the independent-sample t test. A statistically significant difference was defined as *p* < 0.001 for the means of the upper and lower group item scores for all items.

The reliability of the measurement model was also tested using the AVE and CR values for each factor separately. Additionally, to validate concurrency, the AVE values of each construct had to be higher than 0.5, and the CR values were calculated for each construct with values higher than the AVE values. The AVE values of the factors were between 0.50 and 0.74 and the CR values of the factors were between 0.82 and 0.94 during this study (Table 3).

**Table 3:** AVE and CR values for each factor of the BENEFITS-CCCSAT

Factors	1	2	3	4	5	AVE	AVE square root	CR
Perceived meaning of cultural care	1.000					0.50	0.7	0.88
Culturally sensitive communication	-0.110*	1.000				0.74	0.86	0.94
Respect for cultural diversity	0.543**	-0.216**	1.000			0.50	0.7	0.84
Challenges and barriers in providing culturally competent care	-0.189**	0.572**	-0,364**	1.000		0.65	0.81	0.88
Achieving cultural competence	0.404**	-0.288**	0.478**	-0.342**	1.000	0.61	0.78	0.82

*Note.* AVE, average explained variance; CR, composite reliability; **p < 0.001

## Discussion

Studies involving health science students have shown favorable results in terms of cultural competence in different geographical locations [25]. However, there is still room for improvement in the attitudes of students and health professionals towards transcultural practice, as evidenced by the results of other studies [26, 27].

Most currently-available validated tools are the result of specific theoretical models, thus highlighting the uniqueness of each model. However, the BENEFITS-CCCSAT merges the contents and characteristics of most existing tools, thereby providing a new instrument validated in a multicultural context. In this study, the superiority of the measurement tool was obvious.

During this study, the main survey involved 459 undergraduate nursing students after language adaptation [22, 23], content validity [24] and a pilot study that evaluated test-retest reliability [19, 21]. Furthermore, the method of this study was accepted as suitable and in accordance with the literature. A sample size is deemed sufficient for factor analysis when the KMO values are between 0.8 and 1.0. KMO values less than 0.6 indicate that the sampling is not sufficient and that remedial action is necessary [28]. The KMO value of this study was 0.883, indicating that the sample size was sufficient for factor analysis. The statistically significant value of *p* < 0.05 indicated that a factor analysis may be worthwhile for Bartlett’s test of sphericity [28, 29]. During this study, the Bartlett’s values were χ2 = 9683.680 and *p* < 0.01. This finding supported that the sample size and that the correlation matrix of the surveyed items were suitable for factor analysis [30].

As the results of the Varimax rotation showed that a five-factor structure was suitable, the items in this study were categorized into five factors that explained almost 70% of the variance during the analysis. For multifactorial patterns, factors that explained variances of 40–80% are accepted as sufficient [30]; therefore, the contribution of a descriptive factor to the total variance was observed to be sufficient in the current study.

According to the scientific literature, the generally accepted rule is that a Cronbach’s α of 0.6 to 0.7 indicates an appropriate level of reliability. A Cronbach’s α value of 0.8 or more indicates a very good level of reliability [31–33]. In the current study, the BENEFITS-CCCSAT had an overall α value of 0.828 and α values of 0.789 to 0.942 for dimensions. These results show that the items of the tool serve the purpose of measuring the feature that requires measurement. The corrected item correlation value for reliability was more than 0.20. The total correlation score for the items was sufficient [29, 31–33].

The raw scores obtained from the tool are ordered from high to low to determine the discrimination power of the items of the tool. If there is a significant difference between the means of the lower 27% and the upper 27% (*p* < 0.05), then the scale is distinctive in terms of measuring the desired quality [29]. A significant difference was considered when *p* < 0.001 for the means of the lower group and upper group item scores for all items. Based on this principle, the BENEFITS-CCCSAT was distinctive in terms of measuring the desired quality.

### Limitations

Although the tool was developed by researchers from five different countries and cultures and the sample was sufficient, the testing involved undergraduate nursing students studying at two universities in one country. Therefore, further research is needed to evaluate the suitability of the BENEFITS-CCCSAT for different languages and cultures. Furthermore, the BENEFITS-CCCSAT results were not based on objective or observational data; therefore, social desirability bias may be present because of the nature of the instrument.

## Conclusions

The BENEFITS-CCCSAT appears to be a valid and reliable instrument for measuring the cultural sensitivity and cultural competence of nursing students. This can be of great use, especially before attending clinical areas, and can offer both students and faculty reliable information to promote reflective and critical thinking, especially in areas where improvement is needed. The effectiveness of transcultural nursing education can be evaluated using the BENEFITS-CCCSAT. Additionally, the progress of nursing students in transcultural nursing can be monitored. In the future, the validity and reliability of this tool should be retested using studies of graduated nurses so the scale can be applied to groups with different characteristics and attitudes.

## Data Availability

The datasets used and/or analysed during the current study available from the corresponding author on reasonable request.
